# The effects of stimulant medications on the sleepiness curve of young men with Attention-Deficit Hyperactivity Disorder (ADHD)

**DOI:** 10.1186/s12888-026-08011-2

**Published:** 2026-03-24

**Authors:** Iris Haimov, Orrie Dan, Shahar Eisenstein, Kfir Asraf, Ami Cohen

**Affiliations:** Psychology Department, The Center for Psychobiological Research, The Max Stern Yezreel Valley College, Emek Yezreel, Israel

**Keywords:** ADHD, Sleepiness, KSS, Stimulant medications, Sleep deprivation

## Abstract

**Background:**

The present study aimed at investigating the effects of sleep deprivation and stimulant medication (methylphenidate and amphetamine) on subjective sleepiness in young adults with ADHD, compared to individuals without ADHD.

**Methods:**

Fifty-nine young men (age 18–35) of whom 39 were diagnosed with ADHD combined type (ADHD-C) and 20 without ADHD. The participants’ sleep was monitored for 5 days via actigraphy. Subsequently, the participants were kept continuously awake in a controlled environment for 25 h (8amtill 9am the next day). Among the ADHD group, 17 participants were medicated with their regular doses of methylphenidate (*n* = 13) or amphetamine (*n* = 4) at the start of the experiment (08:00 AM) and again at midnight (00:00), while 22 were unmedicated throughout the study. The sleepiness of the participants was assessed every hour by the Karolinska Sleepiness Scale (KSS) in order to obtain the sleepiness curve of both study groups.

**Results:**

Unmedicated ADHD participants reported significantly higher sleepiness throughout the protocol, especially during nighttime and early morning hours. At the end of the 25-hour wakefulness period, their KSS scores were significantly higher than both the control and medicated ADHD groups. No significant difference was found between the medicated ADHD group and controls. Additionally, 88.2% of unmedicated ADHD participants scored above 7 on the KSS (indicating extreme sleepiness), compared to 55% in controls and 36.9% in the medicated ADHD group.

**Conclusions:**

Young adults with ADHD exhibit heightened vulnerability to sleep deprivation, reflected in elevated subjective sleepiness. Stimulant medications effectively attenuate sleepiness in ADHD participants, aligning their alertness levels with those of neurotypical controls. These findings support models of ADHD involving arousal dysregulation and highlight the dual therapeutic role of stimulants in managing both attentional deficits and sleep-related impairments.

**Clinical trial number:**

Not applicable.

## Background

Attention-Deficit/Hyperactivity Disorder (ADHD) is a highly prevalent, chronic neurodevelopmental disorder characterized by persistent, impairing patterns of inattention and/or hyperactivity-impulsivity, affecting an estimated 7.2% of children globally [1, 2]. The disorder is formally recognized by three distinct clinical presentations (subtypes): Predominantly Inattentive Presentation (ADHD-PI), often associated with challenges in executive functions and organizational skills; Predominantly Hyperactive-Impulsive Presentation (ADHD-HI); and the Combined Presentation (ADHD-C), which represents the most common form [3, 4]. The etiology of ADHD is fundamentally multifactorial, underpinned by a highly polygenic architecture; recent large-scale genome-wide association studies (GWAS) have identified 27 independent risk loci, firmly establishing genetics as the primary risk factor [5, 6]. This genetic vulnerability impacts core brain regions, such as the prefrontal cortex, basal ganglia, and cerebellum, and is strongly associated with dysregulation of dopaminergic and noradrenergic systems, thereby supporting the long‑standing dopamine hypothesis, which posits that altered dopaminergic signaling contributes to the cognitive and behavioral manifestations of ADHD [7, 8].

Sleep disturbance is a pervasive comorbidity in Attention-Deficit/Hyperactivity Disorder (ADHD), often contributing to a more severe clinical presentation of ADHD symptoms across the lifespan. Both children and adolescents with ADHD exhibit notable sleep discontinuity, characterized by increased sleep-onset latency and greater nocturnal movements compared to neurotypical peers [9, 10]. This association extends into adulthood, with numerous systematic reviews confirming that adults with ADHD report significantly poorer sleep quality and greater sleep-onset latency across both subjective and objective (actigraphic) measures [11, 12]. Crucially, these nocturnal deficits translate into impaired daytime functioning, manifesting as excessive daytime sleepiness (EDS) or a state of hypoarousal, which is considered by some to be a core physiological feature, particularly in children [13]. Among adults, this daytime sleepiness significantly mediates the relationship between ADHD symptom severity and deficits in cognitive performance, suggesting that sleep-related impairment acts as a key contributing factor to executive dysfunction and overall functional outcomes [14].

The Karolinska Sleepiness Scale (KSS) [15] is a widely utilized rating scale designed to provide a rapid, subjective assessment of an individual’s immediate sleepiness level ranging from extremely alert to very sleepy, fighting sleep. Originally developed and introduced by Åkerstedt and Gillberg [15], the KSS is particularly valued for its high temporal resolution, reflecting moment-to-moment fluctuations in alertness, making it highly effective for measuring sleepiness during sleep deprivation protocols and shift-work simulations [15]. Furthermore, the scale has undergone rigorous validation, demonstrating a robust correlation with objective measures of central nervous system alertness, including electroencephalography (EEG) indicators (e.g., theta/alpha power) and impaired performance on vigilance tasks [16]. Its validity, simplicity, and sensitivity to acute changes in wakefulness have cemented its status as a standard tool in both clinical sleep research and occupational health studies.

In our previous study, we investigated the “sleepiness curve” of young adult males (aged 18–30) with combined-type Attention-Deficit/Hyperactivity Disorder (ADHD-C), who were not receiving pharmacological treated for ADHD, during 25 h of continuous wakefulness. We aimed to determine their vulnerability to fatigue in comparison to neurotypical controls [17]. Although objective baseline sleep measures via actigraphy did not reveal significant differences in total sleep time or efficiency between the groups, the hourly assessments using the Karolinska Sleepiness Scale (KSS) demonstrated a significantly elevated subjective sleepiness curve in the ADHD group throughout the sleep deprivation protocol. Crucially, this difference was most pronounced during the night and early morning hours, specifically between 1:00 a.m. and 9:00 a.m., the period associated with maximal homeostatic sleep drive and circadian misalignment. These findings suggest that young adults with ADHD-C exhibit a lower threshold for subjective fatigue when facing extended wakefulness, lending empirical support to the hypoarousal model by indicating a fundamental difference in central nervous system alertness regulation [17].

Pharmacological treatments are a foundational component of ADHD management across childhood, adolescence, and adulthood, demonstrating both efficacy and tolerability. Stimulant medications, primarily methylphenidate and amphetamines, remain the most effective and commonly prescribed agents, showing robust symptom reduction across age groups [11, 18]. These medications often exert their benefits by enhancing dopaminergic and noradrenergic activity in the brain, mechanisms closely tied to increased wakefulness and alertness throughout the day. For individuals who are unresponsive to or cannot tolerate stimulants, non-stimulant medications such as atomoxetine and guanfacine provide alternative options, albeit with somewhat lower efficacy profiles [11, 18]. In adolescents, pharmacotherapy has been shown to improve not only core symptoms but also broader quality-of-life domains when treatment adherence is maintained [19, 20]. Importantly, medication effects on quality of life appear to vary by age and subtype, with early and individualized intervention showing the greatest benefits. These findings underscore the importance of tailoring pharmacological strategies to developmental stage and patient-specific needs.

The present study aims to examine whether pharmacological treatment for ADHD modulates alertness levels across an extended period of wakefulness in adults. Specifically, the research compares three groups: individuals with ADHD receiving medication, individuals with ADHD not receiving medication, and neurotypical controls without ADHD. Participants in all groups were monitored for 25 consecutive hours of wakefulness under controlled conditions, with hourly assessments of subjective alertness. We hypothesized that the medicated ADHD group would exhibit alertness levels comparable to the control group and significantly higher than the unmedicated ADHD group.

## Methods

### Participants

The present study included 59 male participants aged 18–35 years (Mean: 25.46, SD: 4.37). Thirty-nine participants were diagnosed with ADHD-C, and 20 served as controls without ADHD. Among the ADHD group, 17 participants were medicated with their regular doses of methylphenidate (*n* = 13) or amphetamine (*n* = 4) at the start of the experiment (08:00 AM) and again at midnight (00:00), while 22 were unmedicated throughout the study.

The sample size calculation was based on a small-to-medium effect size (η² = 0.04), using the KSS as the primary outcome measure, with α = 0.05 and a power of 0.95 to detect an effect if present. For a design involving three groups and 25 repeated measures, the minimum required sample size was *N* = 48, meaning at least 16 participants per group.

Inclusion in the ADHD groups required: (a) a formal clinical diagnosis of ADHD made by a licensed neurologist or psychiatrist; (b) endorsement of at least six inattention and six hyperactivity–impulsivity symptoms on the ADHD Rating Scale-IV [21]; and (c) fulfillment of DSM-IV diagnostic criteria for ADHD in the adapted Diagnostic Interview Schedule for Children (DISC-IV) [22]. Participants in the control group met the following criteria: (a) no prior ADHD diagnosis, (b) fewer than four symptoms in either the inattention or hyperactivity–impulsivity subscales, and (c) non-fulfillment of DSM-IV criteria for ADHD based on the clinical interview.

Exclusion criteria for all groups included: (a) presence of any psychopathology according to the Symptom Checklist-90 (SCL-90; Derogatis., 1994) as assessed by a licensed clinical psychologist; (b) employment involving night shifts; or (c) use of medications affecting the central nervous system other than ADHD medication (including sleep medications).

All participants were male to minimize potential variability associated with menstrual cycle effects on sleep quality [23]. The participants were recruited from the general population through advertisements in the social media and received a monetary voucher equivalent to approximately $125 for their participation. Written informed consent was obtained from all participants. The study was conducted in accordance with the Declaration of Helsinki and, thus, the Max Stern Yezreel Valley College Institutional Ethics Review Board approved the complete study protocol (approval number: EMEK YVC 2019-23).

### Measures

**Demographic Questionnaire**: Collected data on age, occupation and health, as well as tobacco smoking status (Yes/No), alcohol use (Yes/No) and medication use.

**ADHD Rating Scale–IV** [21] is an 18-item questionnaire for the assessment of ADHD. The items are based on the symptoms listed in the DSM–IV for ADHD diagnosis, including 9 items assessing attentiveness and 9 items assessing hyperactivity and impulsivity. In the version used in the current study [24], participants were asked to choose whether each described symptom was correct or incorrect with respect to them. The internal consistency (Cronbach’s α) of the attentiveness section and the hyperactivity-impulsivity section of the scale in the current study were 0.82 and 0.87, respectively.

**Structured Clinical Interview**: A modified version of the ADHD module from the DISC [22] was administered in order to determine suitability to the ADHD classification. The modified interview is similar to other interviews that assess ADHD in adulthood [25], and it yields clinician-assessed symptom counts for inattentive and hyperactive-impulsive ADHD symptoms. Internal consistency (Cronbach’s α = 0.86–0.94) was consistent with prior studies.

**The Symptom Checklist–90–Revised** (SCL-90-R [26]): A 90-item self-report inventory assessing psychological symptoms across nine domains. Items were rated on a 0–4 Likert scale, with higher scores indicating greater distress. The internal consistency (Cronbach’s α) of the Hebrew translation of the SCL-90-R was found to be within the range of 0.71–0.85 [27]. An expert clinical psychologist examined the responses of each participant on the SCL-90-R to rule out any psychological disorder. **Pittsburgh Sleep Quality Index** (PSQI [28]): A 18-item self-report questionnaire assessing 7 components of sleep quality (Subjective Sleep Quality, Sleep Latency, Sleep Duration, Sleep Efficiency, Sleep Disturbance, Hypnotic Medication Use, Daytime Dysfunction) over the past month. The seven component scores are then totaled to provide a global PSQI score. The internal consistency (Cronbach’s α) of the Hebrew translation of the PSQI in the current study was 0.73.

**Karolinska Sleepiness Scale** (KSS [15]): A scale measuring subjective sleepiness at a given time. The participant is required to rate his level of sleepiness over the last 10 min on a 9-point Likert scale ranging from 1 (“extremely alert”) to 9 (“extremely sleepy, fighting sleep”).

**Actigraphy**: The actigraph (Mini Motionlogger, Ambulatory Monitoring Inc., New York) is a wrist-worn ambulatory, noninvasive device designed for studies in naturalistic settings with minimal distortions. The actigraph measures wrist movements utilizing a piezoelectric element and translates them into 1-minlong epochs of sleep and wake. To that end, wrist activity levels were sampled at 10-s intervals and summed across 1-min intervals. Actigraphic raw data were translated to sleep measures using the Actigraphic Scoring Analysis program for an IBM-compatible personal computer (W2 scoring algorithm) provided by the manufacturer. Four measures of sleep were obtained: total sleep time (minutes of sleep from intended bedtime to final wake time), sleep onset latency (minutes to fall asleep from bedtime), sleep efficiency (percentage of total sleep time between falling asleep and final awakening), and wake time after sleep onset (WASO; total number of wake minutes after sleep onset). The daily actigraphy data of each subject were averaged over the five days of actigraph use in order to obtain aggregated measures that reliably characterize individuals. The participants were instructed to press a button on the actigraph when they began trying to fall asleep and when they woke up the following morning. The first button-press was used to determine bedtime and the second was used to determine wake time. For the purpose of precise analysis of the actigraph data, over the course of actigraphic recording participants were instructed to complete the Consensus Sleep Diary that included intended bedtime, initial and final wake times, number of awakenings, and lengths of awakenings.

### Procedure

Eligible participants were provided with an actigraph device five days prior to the laboratory session and instructed to wear it for five nights while completing daily evening/morning sleep diaries. They were asked to sleep at least six hours per night to avoid prior sleep deprivation. Members of the research team contacted each participant during the week preceding the experimental trial to ensure adherence to the study instructions, including proper actigraph use and maintaining bedtimes that would allow for at least 6 h of sleep per night.

On the experimental day, participants were collected from their homes at 07:00 AM and transported to the laboratory, where the experiment began at 08:00 AM. Actigraphy data from the five nights preceding the study were reviewed to verify compliance with the sleep requirements. Participants who slept fewer than six hours per night were to be excluded; however, all participants met the minimum sleep criterion, and therefore no exclusions were necessary. After completing baseline questionnaires on demographics and sleep quality (PSQI), participants remained awake for approximately 25 consecutive hours under constant supervision to prevent unintended sleep. Among the ADHD group, 17 participants were medicated with their regular doses of methylphenidate (*n* = 13) or amphetamine (*n* = 4) at the start of the experiment (08:00 AM) and again at midnight (00:00), while 22 were unmedicated throughout the study. Subjective sleepiness was recorded hourly using the KSS. Food and non-caffeinated beverages were provided ad libitum. Upon completion of the 25-hour sleep deprivation protocol, participants were thanked, debriefed, and transported home.

### Data analysis

Data were analyzed using *Jamovi* version 2.5.6. Age and PSQI scores (subjective sleep quality) were compared across groups using one-way Analysis of Variance, (ANOVA). The proportion of alcohol users and smokers were compared across groups using χ2 test. Actigraphy-derived sleep variables (e.g., total sleep time, sleep latency, WASO, sleep efficiency) were compared across groups using the non-parametric Kruskal–Wallis test due to non-normal data distribution. When significant differences emerged, beta regression analyses were conducted to identify predictors of sleep efficiency.

Subjective sleepiness (KSS) was analyzed using a linear mixed-effects model, with group (control/ADHD unmedicated/ADHD medicated) and time (25 h) as fixed effects, and participant as a random effect. Additionally, a logistic regression analysis examined the probability of reporting a KSS score > 7 at the end of the deprivation period as a function of group membership, to assess the impact of ADHD medication on subjective sleepiness under sleep deprivation conditions.

## Results

The study groups did not differ in age or the proportion of tobacco smokers and alcohol users (Table 1). Group differences in sleep variables measured via the PSQI (global scores) and actigraphy were examined (Table 2). No significant differences were found among the three groups in the PSQI scores (subjective sleep quality). In relation to actigraphy-measures sleep variables, no significant differences were found between the groups in total sleep duration [c^2^_(2)_ = 0.39, *p* =.822,*[* sleep onset latency [c^2^_(2)_ = 2.98, *p* =.224], or WASO [c^2^_(2)_ = 3.95, *p* =.138]. However, a significant difference was found in sleep efficiency [c^2^_(2)_ = 7.77, *p* =.020]. Post-hoc analyses revealed that the control group had significantly higher sleep efficiency (M = 0.95, SD = 0.01) compared to both the ADHD group without medication (M = 0.91, SD = 0.01; Z = 2.28, *p* =.044) and the ADHD group with medication (M = 0.90, SD = 0.01; Z = 2.69, *p* =.020). No significant difference was found between the two ADHD groups (Z = 0.43, *p* =.662).

Pearson correlations between the average sleep efficiency, as measured during the week preceding the experimental trial and the level of subjective sleepiness as measured by the KSS at the beginning of the trial (*r* = -.05, *p* =.71) and at its end (e.g., following sleep deprivation; *r* = -.05, *p* =.697) were not significant. Thus, sleep efficiency was not controlled for in subsequent sleepiness analyses.

**Table 1:** Demographic data by group

Measure	Control (*N* = 20	ADHD (*N* = 22)	ADHD + Medication (*N* = 17)	Test Statistic
Age	Mean = 26.62 SD = 4.17	Mean = 24.67 SD = 5.22	Mean = 25.05 SD = 3.39	*F*_(2,53)_ = 1.17 *p* =.315
% Of Smokers	19.05	31.58	5.26	*χ*^2^_(2)_ = 4.34 *p* =.114
% Of Alcohol Users	42.86	47.37	73.68	*χ*^2^_(2)_ = 4.35 *p* =.114

**Table 2:** Subjective sleep (PSQI scores) and actigraphy-based sleep measures by group

Measure	Control (*N* = 20)	ADHD (*N* = 22)	ADHD + Medication (*N* = 17)	Test Statistic
PSQI	Mean = 3.90 SD = 2.90	Mean = 5.58 SD = 3.02	Mean = 5.0 SD = 2.55	*F*_(2,53)_ = 1.76 *p* =.182
Sleep Duration (hours)	Med = 6.95 IQR = 6.8, 7.9	Med = 7.07 IQR = 6.2, 7.6	Med = 7.22 IQR = 6.2, 7.7	*χ*^2^_(2)_ = 0.39 *p* =.822
Sleep Latency (min)	Med = 9 IQR = 7.5, 30	Med = 13.75 IQR = 5.8, 35.2	Med = 7.5 IQR = 5.4, 12.5	*χ*^2^_(2)_ = 2.98 *p* =.224
WASO (min)	Med = 15 IQR = 5, 20	Med = 20.4 IQR = 14.2, 42.6	Med = 17.8 IQR = 9.7, 28.5	*χ*^2^_(2)_ = 3.95 *p* =.138
Sleep Efficiency	M = 0.95 SD = 0.01	M = 0.91 SD = 0.01	M = 0.90 SD = 0.01	*χ*^2^_(2)_ = 7.77 *p* =.020*†

Note: WASO: Wake After Sleep Onset; PSQI: Pittsburgh Sleep Quality Index (subjective sleep quality). *p <.05. Post‑hoc analyses of the sleep‑efficiency data revealed that the control group had significantly higher sleep efficiency than both the ADHD group (Z = 2.28, *p* =.044) and the ADHD + medication group (Z = 2.69, *p* =.020). No significant difference was observed between the two ADHD groups

### Subjective sleepiness (KSS) over time

**Fig. 1:**
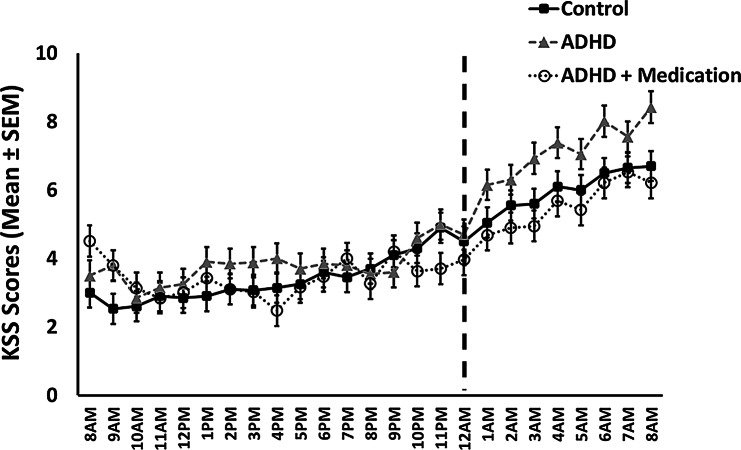
Sleepiness scores as measured by the Karlinska Sleepiness Scale (KSS) each hour during 25 h of the experimental session. The vertical dashed line indicates the timing (midnight) of the second stimulant‑medication administration in the ADHD + medication group

A linear mixed model was used to test the hypothesis that group and time interact to affect subjective sleepiness (KSS) over the 25-hour study period, and particularly following sleep deprivation. Time and group served as independent variables while participants were treated as a random variable. The random variable (σ^2^_Intercept_ = 1.84, 95% C.I.= 1.13,1.65, ICC = 0.48) was significant (LRT = 760.70, *p* <.001). The model explained 66.29% of the variance (R^2^_Conditional_), with the independent variables accounting for 35.18% (R^2^_Marginal_). A significant interaction between time and group was found [F_(48,1392.08)_ = 2.14, *p* <.001]. In addition, there was a significant main effect of time [F_(24,1392.08)_ = 55.79, *p* <.001], but no significant main effect for group [F_(2, 58.99)_ = 1.81, *p* =.171] (see Fig. 1).

Post-hoc comparisons on the KSS scores at the end point of the study (i.e., following 25 h of sustained wakefulness) demonstrated that while there was no difference [*t*_(225.45)_ = -0.77, *p* =.437] between the control group (*M* = 6.70, *SE* = 0.43) and the ADHD + medication group (*M* = 6.21, *SE* = 0.45), the ADHD group (*M* = 8.42, *SE* = 0.45) reported significantly higher sleepiness than both the control group (*t*_(245.48)_ = − 2.70, *p* =.007) and the ADHD + medication group (*t*_(244.96)_ = − 3.43, *p* <.001).

A second linear mixed model tested the hypothesis that from 1:00 AM onward, the ADHD group would report higher sleepiness than the control and the ADHD + medication group, with no difference between the latter two groups. Group was the independent variable, and the participants were the random variable. The random variable (σ^2^_Intercept_ = 2.65, 95% C.I.= 1.34,2.00, ICC = 0.54) was significant (LRT = 221.7, *p* <.001). The model explained 57.83% of the variance (R^2^_Conditional_), with Group accounting for 8.25% (R^2^_Marginal_). A significant group effect was found [_(2,58.95)_ = 4.49, *p* =.015]. Post-hoc comparisons demonstrated that while there was no difference [*t*_(58.60)_ = 0.81, *p* =.419] between the control group (*M* = 6.01, *SE* = 0.38) and the ADHD + medication group (*M* = 5.57, *SE* = 0.39), the ADHD group (*M* = 7.16, *SE* = 0.38) reported significantly higher sleepiness than both the control group (*t*_(59.14)_ = − 2.11, *p* =.039) and the ADHD + medication group (*t*_(59.12)_ = 2.89, *p* =.005).

**Fig. 2:**
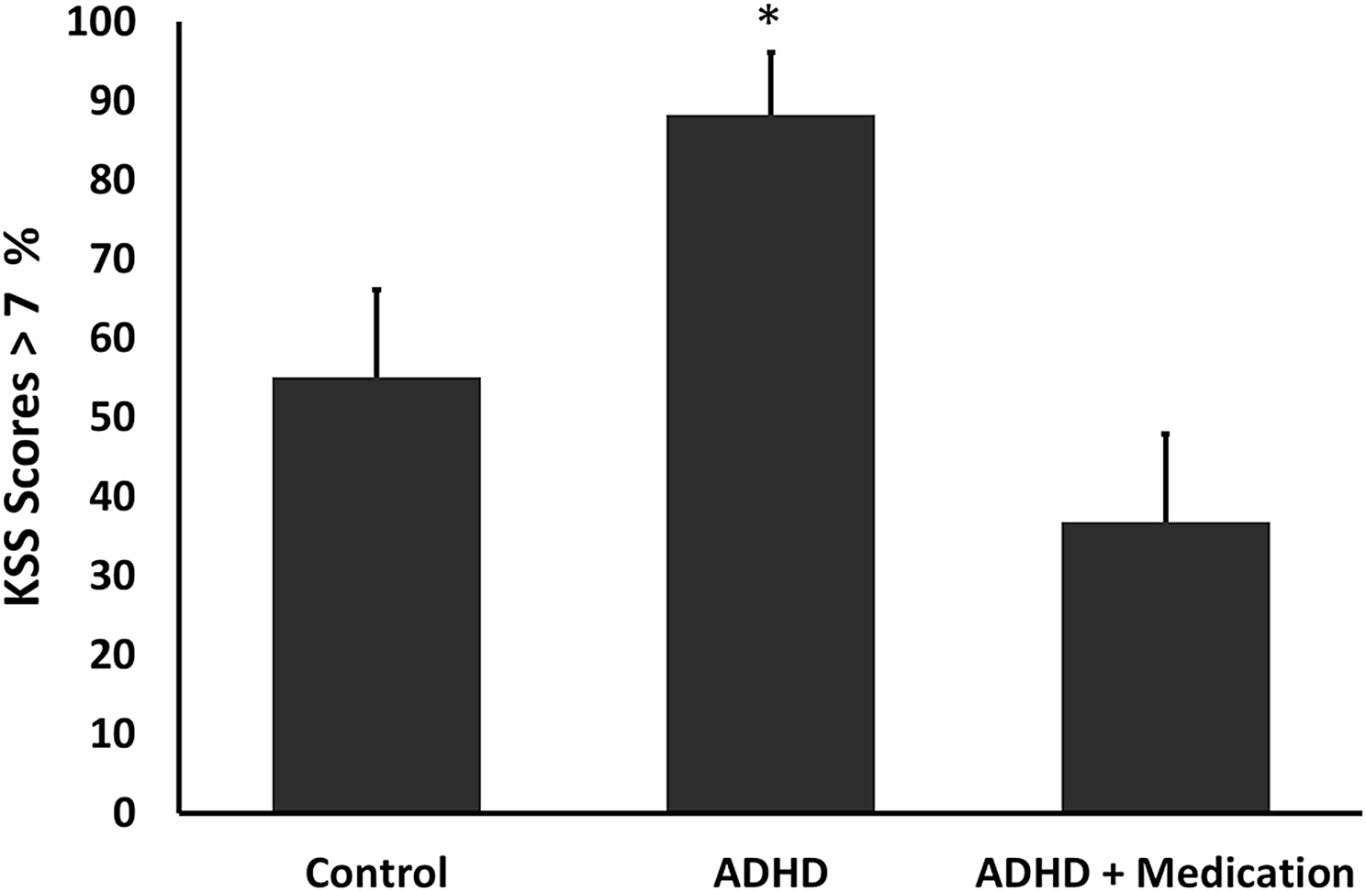
Percentage of participants reporting KSS above 7. Error bars reflect standard error of the proportion in the population. *P <.05 in comparison to the control group

As KSS scores greater than 7 indicate extreme sleepiness with substantial difficulty remaining awake, A logistic regression tested group differences in the proportion of participants with KSS > 7 at the end of the study. The model was significant [*c*^2^_(2)_ = 10.98, *p* =.004, *R*^2^_McFadden_ = 0.0145]. It has been revealed that while the proportion of participants with KSS > 7 at the end of the study was 55.0% in the control group, it reached 88.2% in the ADHD group (see Fig. 2), a difference that was statistically significant (*Z* = -2.07, *p* =.038). In the ADHD + medication group the proportion was 36.9%, significantly lower than ADHD without medication (*Z* = 2.86, *p* =.004), and not significantly different from control (*Z* = 1.13, *p* =.258).

## Discussion

This study investigated the effects of sleep deprivation and stimulant medication (methylphenidate and amphetamine) on subjective sleepiness in young adults with ADHD, compared to individuals without ADHD. Subjective sleepiness was assessed hourly over a 25-hour experimental session using the Karolinska Sleepiness Scale (KSS). The findings support the study’s hypotheses: participants with ADHD exhibited significantly higher levels of sleepiness throughout the experiment, particularly following 25 h of sleep deprivation. Notably, medicated ADHD participants and control participants did not differ significantly in their sleepiness levels.

The observation that unmedicated individuals with ADHD experienced elevated sleepiness during sustained wakefulness, especially overnight and into the following morning, replicates our previous findings [17] and aligns with studies reporting excessive daytime sleepiness in both children and adults with ADHD [29–32]. However, our studies are among the first to systematically compare sleepiness levels throughout the day between individuals with ADHD and the general population. Furthermore, the present study adds novelty by also comparing the sleepiness levels of medicated individuals with ADHD to those of their unmedicated counterparts.

The sleepiness trajectory observed in the control group mirrored patterns reported in prior KSS-based studies conducted in healthy adults [33, 34], with relatively stable levels from morning to evening followed by a gradual increase along the night. In contrast, participants with ADHD showed a significantly steeper rise in sleepiness during the night and subsequent morning. This was further reflected in the proportion of participants scoring above 7 on the KSS the morning after sleep deprivation, 88.2% in the ADHD group versus 55% in the control group, indicating heightened vulnerability to sleep deprivation among individuals with ADHD.

The differences in sleepiness in the current study between the control participants and the non-medicated ADHD participants cannot be attributed to psychiatric comorbidities that are commonly associated with ADHD [35] as individuals suffering from such psychopathologies were excluded from the study. They also cannot be explained by the tendency of ADHD patients to exhibit lower sleep quantity and quality [36–39]. First, the study included only individuals without diagnosed sleep disorders and the participants were instructed to maintain a minimum of six hours of sleep for nights prior to the experimental trial. Second, although average PSQI scores, reflecting subjective sleep quality, were lower in the control group (3.90) than in the ADHD (5.58) and ADHD + medication groups (5.00), these differences did not reach statistical significance. In addition, actigraphy data collected during the 5 nights preceding the experiment revealed that the study groups did not differ in total sleep duration or sleep onset latency. Although sleep efficiency was higher in the control group compared to the unmedicated ADHD group this difference likely did not account for the differences in subjective sleepiness as there were no significant correlation between sleep efficiency and the KSS scores at either the beginning of the experimental trial or its end. These findings are consistent with prior research suggesting that increased sleepiness in ADHD is not solely due to sleep disturbances [29, 40]. Taken together, these findings support the notion that sleep deprivation exacerbates sleepiness in individuals with ADHD, indicating increased vulnerability to fatigue in this population.

Stimulant medications, particularly methylphenidate and amphetamines, are the first-line treatment for ADHD [41]. The current findings demonstrate that these medications not only improve attention and reduce hyperactivity but also normalize subjective sleepiness levels in individuals with ADHD, making them comparable to those of non-ADHD participants. This is consistent with previous research indicating that stimulants can reduce daytime sleepiness [42, 43].

The findings underscore the importance of considering sleep-related factors in both the theoretical understanding and clinical management of ADHD. From a theoretical perspective, these findings support to models that conceptualize ADHD as involving dysregulation in arousal and sleep systems, in addition to cognitive and behavioral symptoms. Pharmacologically, methylphenidate and amphetamine enhance central dopamine and norepinephrine activity by inhibiting their respective transporters [44]. Given their dual efficacy in reducing ADHD symptoms [45, 46] and sleepiness, it is plausible that dysregulation in these neurotransmitter systems underlies both domains [38]. Although the impact of stimulants on sleepiness may be independent of their cognitive effects, prior research has shown a positive correlation between daytime sleepiness and inattentiveness in ADHD [47], suggesting that improvements in attention may be partially mediated by reductions in sleepiness. However, this intriguing hypothesis warrants further empirical investigation.

Clinically, the study highlights the dual role of stimulant medications - not only in improving attention and reducing hyperactivity but also in alleviating daytime sleepiness, particularly under conditions of sleep deprivation. However, it is important to note that the impact of stimulant medication on sleep is complex [48, 49]. Some studies report adverse effects when stimulants are taken late in the day, including increased sleep latency and reduced sleep efficiency [49–52]. Therefore, clinicians should monitor sleep patterns and adjust medication timing and dosage to optimize therapeutic outcomes while minimizing sleep-related side effects.

Interpretation of the present findings should be viewed in light of a few limitations. First, due to constraints imposed by the experimental design, the study sample was limited to young adult males (ages 18–30) in order to minimize variability and enhance statistical power [23]. Consequently, the generalizability of the findings to females and other age groups remains limited. Second, although actigraphy is generally considered a reliable method for obtaining objective sleep-wake data [53], it is not without limitations. Specifically, it has been shown that actigraphy may misidentify quiet, low‑motion wakefulness as sleep [54]. Third, in the current study, ADHD medications were administered at the start of the experiment (08:00) and again at midnight (00:00). This dosing schedule was chosen in light of the relatively short duration of action of these medications and to approximate common clinical practice. However, this dual‑dose design limited our ability to precisely determine the differential contribution of the morning versus midnight dose to the nocturnal sleepiness profile. Future studies employing more refined dosing schedules (for example, directly comparing a single morning dose with a combined morning‑plus‑night regimen) will be necessary to clarify this issue. Another limitation related to medication use is that participants in the ADHD + medication group were treated with either methylphenidate (*n* = 13) or amphetamine (*n* = 4). Although both agents are classified as stimulants, they differ in their pharmacological mechanisms and may exert subtly distinct effects on sleep–wake regulation. Accordingly, direct comparisons of the effects of these medications on wakefulness should be a focus of future research. Fourth, because all ADHD participants in the present study were of the combined subtype (ADHD‑C), the extent to which these findings generalize to individuals with other ADHD subtypes remains uncertain. In addition, as in most ADHD research, individuals with psychiatric comorbidities were excluded. Although this approach enhances internal validity by isolating ADHD‑specific effects and minimizing confounding influences, it substantially limits the generalizability of the results to the broader and clinically heterogeneous ADHD population. Fifth, the experiment was conducted in a controlled laboratory setting, which may not reflect real-world conditions where environmental factors vary widely. Finally, the extreme sleep deprivation protocol (25 h) represents an atypical scenario that may elicit exaggerated behavioral and cognitive responses.

Future studies should include female participants and examine potential modulatory effects of the menstrual cycle. Moreover, future research should broaden the sample to include individuals with different ADHD subtypes as well as those with common psychiatric comorbidities, in order to test the generalizability of the present findings. In addition, future studies would benefit from employing less extreme sleep‑restriction protocols (e.g., allowing 4–5 h of sleep per night), which may better reflect real‑world sleep deprivation patterns.

## Conclusions

This study provides empirical support for the heightened vulnerability of individuals with ADHD to the effects of sleep deprivation on sleepiness. The stimulant medications methylphenidate and amphetamine were shown to effectively attenuate sleepiness in ADHD participants, aligning their wakefulness levels with those of non-ADHD controls. These findings highlight the dual therapeutic role of stimulants in managing both attentional deficits and sleep-related vulnerabilities in ADHD.

## Data Availability

Data is available upon reasonable request from the corresponding author.

## Abbreviations

ADHD: Attention-Deficit Hyperactivity Disorder
ADHD - C: Combined Type Attention-Deficit Hyperactivity Disorder
ADHD - PI: Predominantly Inattentive Attention-Deficit Hyperactivity Disorder
ADHD - HI: Predominantly Hyperactive-Impulsive Attention-Deficit Hyperactivity Disorder
DISC: Diagnostic Interview Schedule for Children
DSM-IV: Diagnostic and Statistical Manual of Mental Disorders, 4th Edition
EEG: Electroencephalography
EDS: Excessive Daytime Sleepiness
KSS: Karolinska Sleepiness Scale
PSQI: Pittsburgh Sleep Quality Index
SCL-90: Symptom Checklist-90
WASO: Wake After Sleep Onset
